# The Role and Mechanism of Probiotics Supplementation in Blood Glucose Regulation: A Review

**DOI:** 10.3390/foods13172719

**Published:** 2024-08-27

**Authors:** Xinyu Shen, Chunmin Ma, Yang Yang, Xiaofei Liu, Bing Wang, Yan Wang, Guang Zhang, Xin Bian, Na Zhang

**Affiliations:** College of Food Engineering, Harbin University of Commerce, Harbin 150028, China; ssssxy218@126.com (X.S.); chunmin_ma@163.com (C.M.); 13766948050@163.com (Y.Y.); liuxiaofei72@163.com (X.L.); iceking85@163.com (B.W.); wangyan123456@163.com (Y.W.); rczkzg@163.com (G.Z.); bianbian1225@163.com (X.B.)

**Keywords:** probiotics, blood glucose regulation, type 2 diabetes mellitus, gut microbiota, insulin resistance, intestinal flora

## Abstract

With economic growth and improved living standards, the incidence of metabolic diseases such as diabetes mellitus caused by over-nutrition has risen sharply worldwide. Elevated blood glucose and complications in patients seriously affect the quality of life and increase the economic burden. There are limitations and side effects of current hypoglycemic drugs, while probiotics, which are safe, economical, and effective, have good application prospects in disease prevention and remodeling of intestinal microecological health and are gradually becoming a research hotspot for diabetes prevention and treatment, capable of lowering blood glucose and alleviating complications, among other things. Probiotic supplementation is a microbiologically based approach to the treatment of type 2 diabetes mellitus (T2DM), which can achieve anti-diabetic efficacy through the regulation of different tissues and metabolic pathways. In this study, we summarize recent findings that probiotic intake can achieve blood glucose regulation by modulating intestinal flora, decreasing chronic low-grade inflammation, modulating glucagon-like peptide-1 (GLP-1), decreasing oxidative stress, ameliorating insulin resistance, and increasing short-chain fatty acids (SCFAs) content. Moreover, the mechanism, application, development prospect, and challenges of probiotics regulating blood glucose were discussed to provide theoretical references and a guiding basis for the development of probiotic preparations and related functional foods regulating blood glucose.

## 1. Introduction

With the improvement of people’s living standards, a series of changes in dietary structure and lifestyle have occurred, leading to a rapid increase in the prevalence of metabolic diseases. Globally, the number of people with diabetes is expected to be 643 million by 2030 and 783 million by 2045 [1]. Diabetes mellitus is divided into three categories: type 1 diabetes mellitus (T1DM), type 2 diabetes mellitus (T2DM), and gestational diabetes mellitus (GDM), with the most significant number of patients suffering from T2DM, accounting for about 90% of the total number of diabetic patients, which has now become a major global crisis [2,3]. Diabetes-related complications such as macrovascular lesions, microangiopathy, and tumors have become the leading cause of death and disability among diabetic patients in the country. Therapeutic measures at this stage cannot cure diabetes but can delay the onset and mitigate complications [4]. Currently, insulin therapy is effective but costly, and IDF data show that there are about 450 million people with diabetes worldwide, and the annual treatment cost is as high as USD 670 billion. There are many hypoglycemic drugs such as metformin, miglitol, acarbose, and glyburide, but each has different toxicity and side effects such as hypoglycemia, body mass increase, edema, nausea, etc. [5,6]. Therefore, most of the current research on T2DM has focused on exploring novel therapeutic approaches without toxic side effects.

**Figure 1:**
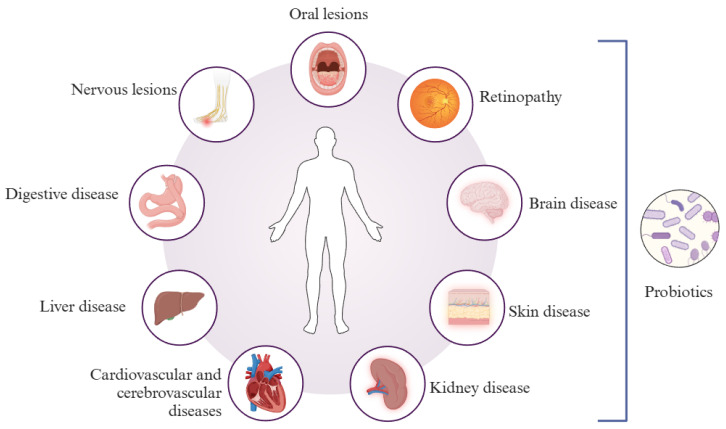
Probiotics may relieve the symptoms of T2DM.

Probiotics are a class of active microorganisms beneficial to the host. They can effectively colonize the human intestinal tract, improving the intestinal microecosystem when ingested in certain quantities, thus regulating the body’s intestinal flora. Probiotic therapy has good application prospects in disease prevention and remodeling of intestinal health due to its low cost, high safety, and reliability [7,8]. According to statistics, the global market of probiotic foods, dietary supplements, and probiotic raw materials is growing at 15% to 20% per year. Probiotics and their products have been shown to improve barrier function, reduce inflammation levels, and enhance immunomodulation. They are considered economical and safe alternatives for treating chronic diseases [9,10,11,12]. Some probiotics and their main functions are shown in Table 1. In recent years, some studies have found that intestinal flora and homeostasis play an essential role in the treatment of T2DM, and progress has been made in both clinical studies and scientific research. For example, the results of clinical studies have shown that probiotics can regulate blood glucose in patients with T2DM and that probiotics reduce blood glucose and inflammatory responses by improving intestinal flora, leaky gut, and endotoxemia in patients with T2DM [13,14]. Probiotics in scientific studies improved T2DM by mediating the gut microbial-SCFA-hormone/inflammatory pathway in mice [15]. Therefore, probiotics and their products will play an essential role in improving the health of T2DM patients in the future (Figure 1).

**Figure 2:**
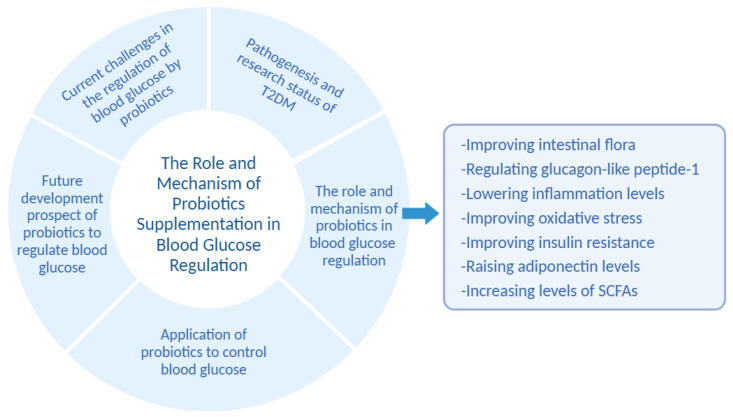
Schematic representation of the topic.

This paper reviews the mechanism of action, application, development trend, prospects, and challenges of probiotics in regulating T2DM, with a view to providing a theoretical basis for the development of microecological agents for regulating blood glucose (Figure 2).

## 2. Methods

**Figure 3:**
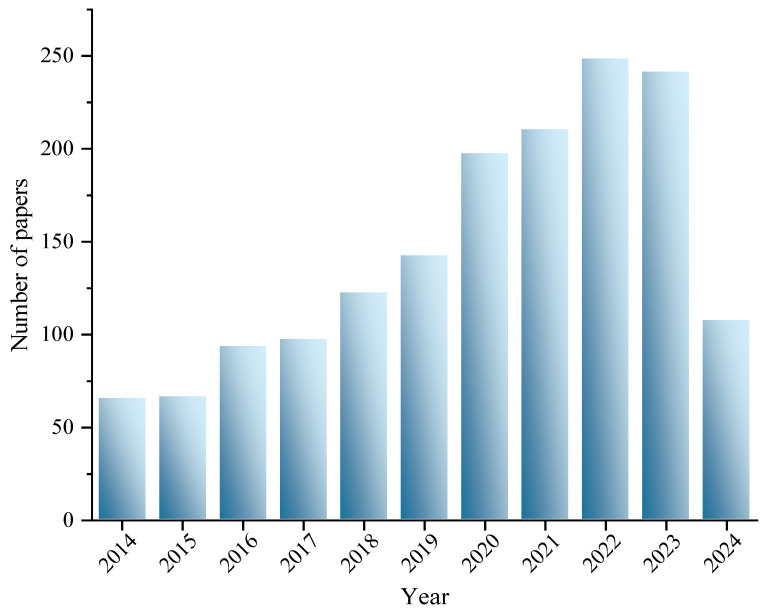
Number of published papers (2014–2024).

**Figure 4:**
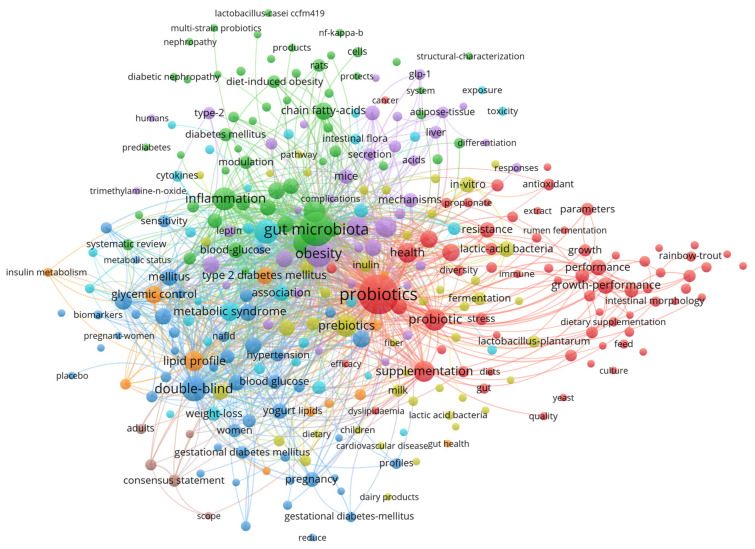
Keyword co-occurrence network diagram of published articles that focused on probiotics and blood glucose (2014–2024).

This review was conducted by electronically searching the literature using the Web of Science. A total of 1599 papers that focused on probiotics and blood glucose from 2014 to 2024 were collected. The publication trend of the review topic keywords is shown in Figure 3. The keywords co-occurrence network illustrated the progress in research on the role and mechanism of probiotics supplementation in blood glucose regulation (Figure 4).

## 3. Pathogenesis and Research Status of T2DM

**Figure 5:**
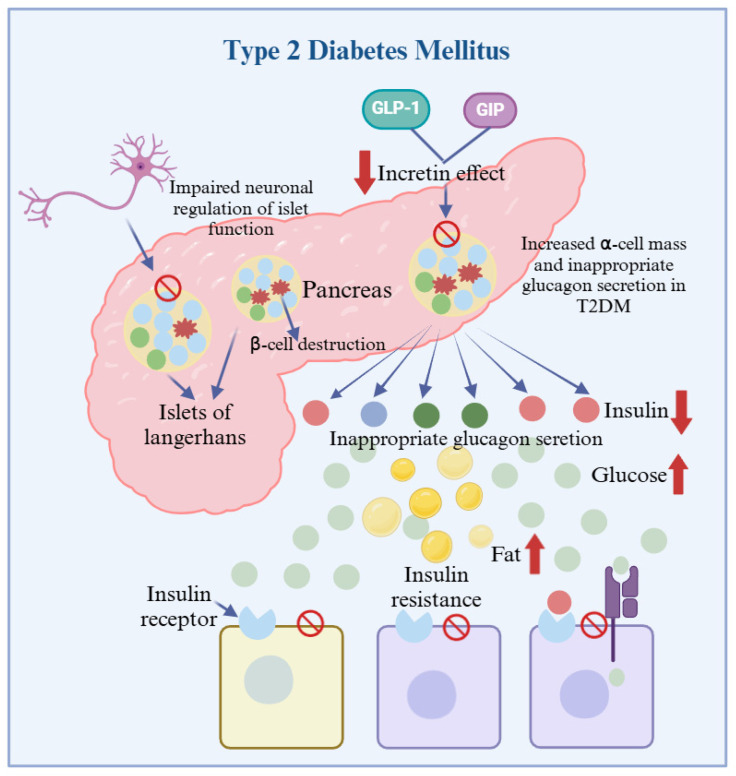
The pathogenesis of T2DM. The function of surviving cells is impaired due to the apoptosis of islet β-cell. This significantly reduces the level of insulin circulating in the blood. In addition, peripheral tissue insulin resistance impairs insulin action, and reduced insulin levels and action can lead to hyperglycemia and hyperlipidemia.

As we all know, diabetes is a chronic metabolic disease that occurs when the body cannot effectively use or produce insulin. The typical feature of diabetes mellitus is that the body cannot maintain normal blood sugar levels, mainly manifested as irritable thirst, excessive eating, polyuria, weight loss, and so on [43]. This chronic metabolic disorder has become a global health problem due to the severe harm it causes to human health [44]. Accelerated urbanization, aging, and lifestyle changes have led to an increase in the rate of obesity, which in turn increases the prevalence of diabetes mellitus and cardiovascular diseases [45]. T2DM is considered as an intestinal disorder that predominates in diabetic patients and is mainly caused by disturbances in glucose metabolism regulated by pancreatic β-cells [46]. Insulin is a peptide hormone secreted by the pancreas by the β-cells which regulates the uptake of glucose in the corpuscular circulation by activating hepatocytes and myocytes as a source of energy or by storing it in the form of glycogen in hepatocytes or skeletal muscle cells. Due to insulin resistance, reduced insulin receptor function and instability of pancreatic β-cells result in the inability of the cells to take up blood glucose, which in turn leads to T2DM [47] (Figure 5). In addition to this, during the formation as well as development of diabetes mellitus, dysbiosis of intestinal flora and endotoxins produced by harmful flora further trigger inflammatory response, oxidative stress, and destruction of pancreatic β-cells leading to T2DM and complications.

T2DM also leads to many complications in addition to the typical symptoms, such as heart disease, stroke, kidney failure, amputation, loss of vision, nerve damage, and increased risk of premature death, with cardiovascular disease being the leading cause of morbidity and mortality in diabetic patients [48,49,50]. In recent years, patients with T2DM have had an increased risk of death. These complications seriously affect the quality of life of patients and increase economic and family stress, making the treatment and prevention of diabetes urgent. The most effective therapeutic measure at this stage is insulin, but the cost of insulin therapy is high, and only about 50% of patients with T2DM receive the required insulin therapy [51]. Moreover, different hypoglycemic agents are tolerated differently (in terms of glycemic control) and have some side effects. In addition to increasing exercise, paying attention to diet, and keeping a comfortable mood, there has been some progress in the research on the use of probiotics to regulate T2DM. However, it is not without side effects.

## 4. The Role and Mechanism of Probiotics in Blood Glucose Regulation

**Figure 6:**
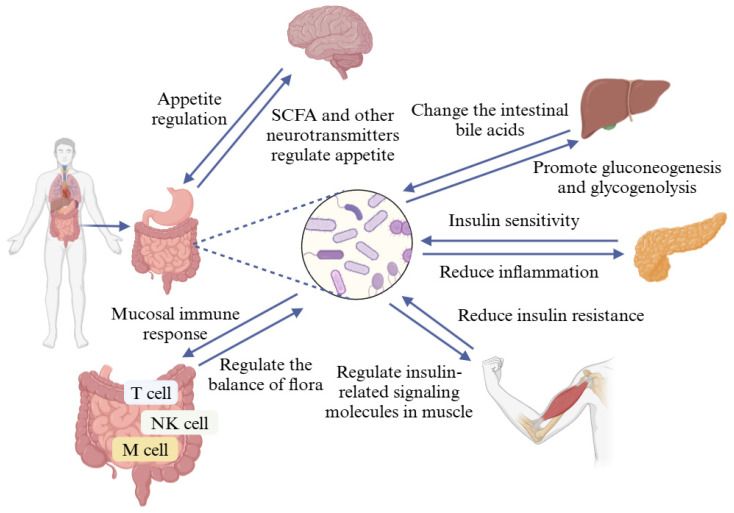
Probiotics regulate blood glucose in a variety of ways. These include regulating the balance of intestinal flora, intestinal immunity, microbe-gut-brain axis, microbe-gut-liver axis, and so on.

Some studies have shown that probiotics regulate blood glucose by regulating intestinal flora balance, intestinal immunity, microbial-gut-brain axis, microbial-gut-hepatic axis, and other pathways (Figure 6) [52,53]. Research on the mechanism of probiotic action on host blood glucose regulation is gradually deepening, and the existing mechanisms of action may include the following pathways: (1) Probiotics can form a biological immune barrier with intestinal mucosa to maintain the balance of intestinal flora [54]. (2) Probiotics can reduce insulin resistance and repair oxidative damage to pancreatic β-cells to regulate blood glucose [55]. (3) Probiotics attach to the intestinal mucosa through adhesins on their surface, constituting a biological barrier and releasing immune factors, thus enhancing the body’s immune function [56]. (4) Probiotics may reduce oxidative stress by decreasing chronic low-grade inflammation [57]. (5) Probiotics may increase autonomic activity and regulate the activity of enzymes related to glucolipid metabolism [58]. (6) Probiotics can produce substances such as bacteriocins, which protect the pancreatic islets, promote the function of pancreatic islets, and inhibit α-glucosidase, playing a key role in regulating blood glucose [59]. (7) The peptides produced during the proliferation of probiotics can inhibit the activity of postsynaptic neurons through the brain-gut axis and improve metabolism [60].

### 4.1. Probiotics Regulate Blood Glucose by Improving Intestinal Flora

The gut microbiota is widely recognized as one of the most important components in maintaining homeostasis. It is closely associated with health and disease in humans and other mammals, and many diseases are accompanied by disorders of the gut microbiota [61]. More and more studies have shown that the development of T2DM is related to host genetics and environmental factors, and intestinal flora, as a significant environmental factor, is closely associated with the occurrence and development of T2DM [62]. Probiotics can colonize the human intestinal tract, improve human intestinal flora, regulate metabolism, and maintain the balance of the intestinal system. Probiotics alleviate and treat diabetes by improving the intestinal barrier function, changing the intestinal flora, increasing the content of SCFAs, decreasing oxidative stress and antioxidant effects, inhibiting the activity of enzymes related to glucose absorption, increasing the activity of bile salt hydrolase, and absorption or adsorption of cholesterol. The parts are interrelated and work together to achieve anti-diabetic efficacy [63,64,65]. Probiotic complexes reduce *E. coli* and lipopolysaccharide levels and improve intestinal barrier function by increasing levels of SCFA-producing bacteria and SCFAs [66]. Park et al. [67] found that treatment with *Lactobacillus flexneri* HY7601 and *Lactobacillus plantarum* KY1032 resulted in reduced body weight gain and changes in the intestinal flora of obese mice fed a high-fat diet. The probiotic blend, VSL#3, has been shown to inhibit weight gain and insulin resistance by altering the composition of the gut flora [68]. The combination of probiotics has a more pronounced effect on blood glucose regulation by improving the intestinal SCFA-producing flora, which in turn plays a role. Therefore, probiotics can have a preventive effect on the occurrence and development of metabolic diseases such as diabetes by improving the composition of intestinal flora, increasing beneficial bacteria, and inhibiting harmful bacteria.

### 4.2. Probiotics Regulate Blood Glucose by Regulating Glucagon-like Peptide-1

**Figure 7:**
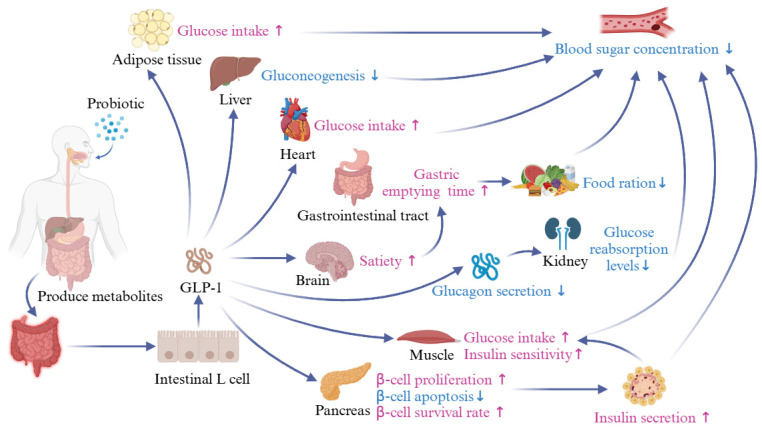
Probiotics relieve diabetes by stimulating GLP-1 secretion. Probiotics can affect the metabolites of intestinal flora by regulating the structure of intestinal flora, activating the metabolic pathway in the host body, and stimulating the secretion of GLP-1 by intestinal L-cells. This alleviates T2DM and enhances the antioxidant capacity of host cells by increasing the content of peroxide dismutase in the body, which helps to clear free radicals and reduce the occurrence of T2DM complications.

Glucagon-like peptide-1 (GLP-1) is a peptide composed of 31 amino acids, which is an enteric hypoglycemic hormone. When the human body eats, intestinal probiotics can utilize nutrients related to their metabolism to produce a variety of metabolites and stimulate the secretion of GLP-1 from intestinal L-cells, which helps to maintain blood glucose balance in the body, reduce food intake, inhibit obesity, and alleviate T2DM among other things [69,70]. Long-term obesity not only destroys the function of pancreatic β-cells and leads to abnormalities in glucose and lipid metabolism but also increases the synthesis of Dipeptidyl peptidase-IV (DPP-IV) in the body and promotes the degradation of GLP-1 [71,72]. Therefore, diet-induced obesity may be a pre-sign of T2DM. Probiotics can influence the metabolites of the intestinal flora to activate metabolic pathways in the host and stimulate the secretion of GLP-1 from intestinal L-cells, which in turn alleviates T2DM and enhances the antioxidant capacity of host cells by increasing the levels of peroxide dismutase in vivo. This contributes to the scavenging of free radicals in vivo and reduces the incidence of complications from T2DM [73,74]. Figure 7 demonstrates that probiotic bacteria stimulate GLP-1 secretion in host cells by producing metabolites to stimulate the secretion of GLP-1 after the mitigating effect on T2DM. When the nutrients ingested by the body from the outside stimulate the signaling molecules on the intestinal cells, the intestinal L-cells will secrete and release GLP-1, which can repair the function of pancreatic β-cells. While doing this, it can also stimulate pancreatic β-cells to divide, gradually restoring their number and promoting insulin secretion back to the average level [75]. However, the gut is not the only source of GLP-1. It is also secreted in the brain. Recent studies have found that strains such as *Lactobacillus paracasei* 1F-20, *Lactobacillus fermentum* F40-4, and *Bifidobacterium animalis* subsp. *paracasei* promote the secretion of GLP-1 and peptide YY (PYY) by up-regulating the glucagon gene (GCG) and PYY genes in stanniocalcin-1 (STC-1) cells. Their metabolites can be regulated by peroxisome proliferator-activated receptor-α (PPARα), sterol regulatory element binding protein-1C (SREBP-1C), patatin-like phospholipase domain containing 3 (PNPLA3), and other regulatory genes reaching the liver to improve lipid accumulation and increase glucose uptake by up-regulating PI3K/AKT activity to restore the insulin signaling system [76]. Acetic acid, propionic acid, and butyric acid are SCFAs that play important probiotic roles in the human body [77]. Studies have shown that fecal levels of acetic acid and butyric acid were significantly elevated after fecal transplantation interventions, which led to activation of the GLP-1 pathway by SCFAs and elevated GLP-1 protein expression in colonic tissues, thereby ameliorating glycolipid disorders [78]. In the future, by exploring the mechanism of action of probiotics and their metabolites on host intestinal cells and regulating their continuous production of GLP-1, we can further clarify the remission effect of probiotics on T2DM and finally provide universal, safe, and effective remission for T2DM patients and promote human health.

### 4.3. Probiotics Regulate Blood Glucose by Lowering Inflammation Levels

T2DM is associated with elevated levels of pro-inflammatory cytokines, chemokines, and inflammatory proteins. Therefore, low-grade systemic inflammation is believed to play a vital role in the development of T2DM and its associated complications [79,80]. The results of the latest clinical trial studies have also shown that the levels of interleukin-6 (IL-6), tumor necrosis factor-α (TNF-α), and interleukin-1β (IL-1β) are significantly higher in T2DM patients than in normal subjects [81]. Studies have shown that diabetic patients have increased intestinal permeability, which correlates with the persistently low level of chronic inflammation present in their intestines, and that the intestinal mucus layer serves as an important barrier that prevents intestinal bacteria from invading the mucosa and causing inflammation [82,83]. Probiotics may ameliorate inflammation by exerting a positive effect on the dysfunction of the epithelial cells and mucosal immune system that form the basis of inflammation. Probiotics alleviate the body’s symptoms in patients with prediabetes and T2DM by reducing inflammation and modulating immunity [84,85]. Amar et al. [86] in their study of the effects of probiotics on bacterial translocation and glucose metabolism in high-fat diet-induced diabetic mice, found that *Bifidobacterium animalis Lactobacillus* 420 application was able to alter the bacterial translocation in the early stages of diabetes and reduce cytokines in tissues TNF-α, IL-1β, plasminogen activator inhibitor-1 (PAI-1), and IL-6 expression. *Lactobacillus plantarum* JY039 extracellular polysaccharide and *Lactobacillus paracasei* JY062 alleviated T2DM by balancing pro-inflammatory factor IL-6, TNF-α, and anti-inflammatory factor Interleukin-10 (IL-10) to reduce inflammation [87]. In addition, probiotics can maintain the stability of the intra-immune environment by balancing pro-inflammatory and anti-inflammatory immune responses, in which SCFAs play an important role in regulating T-cell function and exerting anti-inflammatory effects. The effects of probiotics on inflammatory pathways, weight gain, and glucose metabolism in animals are primarily attributed to the production of SCFAs, which promote the generation and differentiation of regulatory T-cells by directly activating G-protein-coupled receptors and inhibiting histone deacetylase which are potent anti-inflammatory factors. The inflammatory response is alleviated by inhibiting the release of pro-inflammatory factors in lamina propria macrophages [88,89,90].

### 4.4. Probiotics Regulate Blood Glucose by Improving Oxidative Stress

Oxidative damage and antioxidant capacity play essential roles in the pathogenesis of diabetes. Hyperglycemia can directly cause reactive oxygen species (ROS) to increase, and these oxygen free radicals induce oxidative stress, which in turn damages the endogenous antioxidant defense system [91,92,93]. The intake of probiotics can reduce markers of inflammation and oxidative stress, and improve blood glucose and insulin metabolism [94]. Zhang et al. [95] reported that the effect of probiotics on glucose metabolism could be achieved by reducing oxidative stress. Yadav et al. [96,97] showed that probiotics can improve the antioxidant content of glutathione, superoxide dismutase, catalase, and glutathione peroxidase in diabetic rats by inhibiting lipid peroxidation, thereby reducing oxidative damage, increasing insulin secretion, reducing glycosylated hemoglobin level, and reducing intestinal absorption of glucose. This can restore blood glucose to normal levels and ease the development of T2DM.

### 4.5. Probiotics Regulate Blood Glucose by Improving Insulin Resistance

Probiotics can improve insulin sensitivity in patients. The binding of SCFAs produced by them to their receptors can significantly reduce fasting blood glucose, fasting plasma insulin, and insulin resistance index levels. In addition, mixed probiotic supplementation can reduce hepatic transaminases and insulin resistance levels, among others [92,98]. Siebler et al. [99] demonstrated that oral administration of *Bifidobacterium bifidum* reduced intestinal endotoxin concentration, improved glucose tolerance, and alleviated insulin resistance in an animal model, thereby regulating blood glucose.

### 4.6. Probiotics Regulate Blood Glucose by Raising Adiponectin Levels

Adiponectin is an endogenous bioactive polypeptide or protein secreted by adipocytes, and its level is positively correlated with insulin, which can reflect the efficacy and prognosis of T2DM to a certain extent [100]. Compared with healthy people, adiponectin levels in T2DM patients are reduced, and the reduction is more obvious in T2DM patients with complications such as atherosclerosis [101]. Adiponectin improves insulin resistance and protects β-cells by increasing insulin sensitivity. Clinically, it shows hypoglycemic and anti-inflammatory potential, alleviating insulin resistance and enhancing glucose metabolism, which are therapeutic targets for diabetes [102,103].

### 4.7. Probiotics Regulate Blood Glucose by Increasing Levels of SCFAs

**Figure 8:**
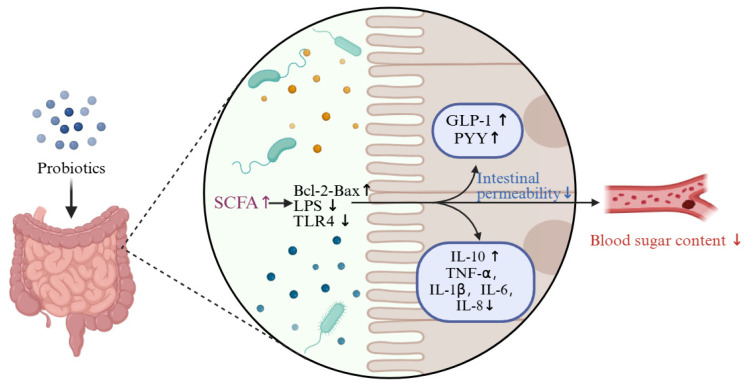
Probiotics relieve blood glucose levels by regulating SCFAs. SCFAs can induce L-cells to secrete GLP-1 and PYY, and promote insulin secretion to relieve T2DM. In addition, SFCAs reduce the level of inflammatory factors through intestinal epithelial cells, thereby reducing blood sugar content.

By regulating the intestinal microenvironment, probiotics can increase the abundance of SCFA-producing bacteria and inhibit the growth of other pathogenic bacteria such as *Escherichia coli* in the intestine, thereby increasing the content of SCFAs in the intestine [104,105]. This can help diabetic patients maintain blood glucose balance and effectively relieve T2DM (Figure 8). SCFAs, including butyric acid, acetic acid, and propionic acid, are produced when gut bacteria ferment dietary fiber. SCFAs have a profound effect on insulin sensitivity and energy metabolism, altering the levels of several intestinal peptides involved in glucose metabolism, intestinal barrier function, and energy homeostasis. For example, butyric acid and propionic acid can inhibit weight gain in obese mice induced by high-fat diets, and acetic acid can reduce food intake in healthy mice [106,107]. The influence of probiotics on metabolic diseases may be partly due to the metabolic regulation of their metabolites—SCFAs and bile acids (BAs). In addition to providing energy substances for the body, SCFAs also play an essential role in regulating insulin sensitivity and energy metabolism [108]. The effects of probiotics on inflammatory pathways, weight gain, and glucose metabolism in animals are primarily attributable to SCFA production [109]. It has been found that SCFAs directly bind to free fatty acid receptor 2 (FFAR2) in mouse’s white adipose tissue as a signaling molecule, inhibiting insulin signal transduction in adipose cells, thereby inhibiting fat accumulation and increasing energy consumption in the liver and muscle [110]. On the other hand, SCFAs as an essential nutrient in the intestinal mucosa promote the growth and differentiation of intestinal epithelial cells and up-regulate the expression of the intestinal tight junction protein gene and proglucagon glucagon-like peptide-2 (GLP-2) gene in intestinal L-cells, thus strengthening the tight junction between intestinal epithelium, reducing intestinal permeability, and improving intestinal barrier function. This reduces bacterial translocation and endotoxemia, thereby controlling obesity and T2DM [111].

## 5. Application of Probiotics to Control Blood Glucose

Currently, in experimental and clinical studies, probiotics may regulate blood glucose in T2DM through a combination of the following pathways: production of substances such as bacteriocins, decreasing inflammation, regulating the intestinal flora, increasing the content of SCFAs, enhancing immunity, and improving insulin resistance. In animal models (Table 2), probiotics can lower blood glucose and prevent damage to pancreatic β-cells by improving inflammation. *Lactobacillus* and *Bifidobacterium* have been shown to improve glucose tolerance and insulin resistance, and *Bifidobacterium* spp. can improve glucose homeostasis in mice induced by a high-fat diet [112,113]. In clinical studies (Table 3), probiotic intervention studies have revealed positive effects on glucose metabolism. Among them, the hypoglycemic effects of *Lactobacillus* and *Bifidobacterium* have been demonstrated in several studies [114,115].

In addition, in recent years, consumers have increasingly favored probiotic preparations and functional foods containing probiotics that improve gastrointestinal health and other functions in the market. Due to the advantages of mild hypoglycemic effect, stable nature, and remarkable effect, probiotics for blood glucose regulation have broad market application prospects. Fruits and vegetables are rich in dietary fiber, vitamins, and various phytochemicals. Dietary modification based on fruits and vegetables is undoubtedly an important direction for the prevention and treatment of T2DM and other chronic diseases. Fermented foods can provide us with more probiotics and prebiotics than other types of supplements. Carrots contain hypoglycemic substances and are a good dietary supplement for diabetics. Xiong et al. [116] prepared fermented carrot juice using *Lactobacillus plantarum* NCU116, which has a hypoglycemic function. Carrot juice fermented with probiotics can not only preserve the fermented flavor, but also meet the basic requirements of modern human healthcare. Johansson et al. [117] applied *Lactobacillus reuteri* 180, which has a hypoglycemic effect on fermented fruit juice. In addition, probiotics can also cooperate with drugs or traditional Chinese medicine to play a role in lowering blood glucose. Studies have found that the combination of probiotics and metformin can enhance the hypoglycemic effect of metformin by regulating intestinal flora and up-regulating intestinal SCFAs [118]. Jang et al. [119] showed that fermented red ginseng with oral probiotics could reduce fasting blood glucose, improve glucose tolerance, and alleviate symptoms of diabetic mice. Flavonoids, alkaloids, polysaccharides, terpenes, and polyphenols in probiotics-fermented fruit juice have an auxiliary hypoglycemic effect.

**Table 2:** Experimental study on the effect and mechanism of probiotics on diabetes mellitus.

Probiotics	Animal Model	Dosage	Duration	Results	Reference
*Lactobacillus plantarum* HAC01	STZ-induced C67BL/6J mice, T2DM	1 × 10^9^ CFU/mL	10 weeks	Insulin-positive β-cells area of the islet ↑ FBS, HbA1c, OGTT and HOMA-IR ↓	Lee et al. [120]
*Lactiplantibacillus plantarum* Y15	STZ-induced C57BL/6J mice, T2DM	3 × 10^8^ CFU/mL	6 weeks	Proinflammatory factors and LPS ↓ SCFA-producing bacteria ↑ Regulated the expression of genes related to inflammation and insulin signaling pathways	Liu et al. [121]
*Lactobacillus gasseri*	Western diet–induced C57BL/6J mice, T2DM	1 × 10^9^ CFU/mL	8 weeks	Serum glutathione and bilirubin ↑ Blood glucose, blood lipids ↓	Rodrigues et al. [122]
*Lactobacillus plantarum* CGMCC 8198	High fat diet–induced Kunming mice, T2DM	0.2 mL/10 g	8 weeks	Harmful bacteria, blood glucose, and blood lipids ↓ Immunity ↑	Jiang et al. [123]
*Akkermansia muciniphila*	STZ-induced SD rats, T2DM	1 × 10^10^ CFU/mL	4 weeks	HDL-C ↑ Liver glycogen, plasminogen activator inhibitor-1, TNF-α, LPS, malondialdehyde, GLP-1 ↓	Zhang et al. [124]
*Lactobacillus reuteri* GMNL-263	STZ-induced Wistar rats, T2DM	1 × 10^9^ CFU/mL	4 weeks	Activate the IGF1R cells’ survival pathway ↑ Cells apoptosis via the IGF1R survival pathway in diabetic rats ↓	Koay et al. [125]
*Lactobacillus sakei* Probio-65 and *Lactobacillus plantarum* Probio-093	High fat diet–induced C57BL/6J male mice, T2DM	0.25 mg/g/day	8 weeks	α-glucosidase, α-amylase, blood glucose, and body weight ↓ Regulated the intestinal flora	Gulnaz et al. [126]
*Lactobacillus fermentum* TKSN041	STZ-induced Wistar male rats, T2DM	—	—	Blood glucose, tissue damage; body weight, blood lipids, and inflammation levels ↓	Zhou et al. [127]
*Lactobacillus fermentum* MCC2759 and *Lactobacillus fermentum* MCC2760	STZ-induced Wistar rats, T2DM	1 × 10^9^ CFU/mL	12 weeks	OGTT, Insulin, IL-10, ZO-1, GLP-1 ↓	Archer et al. [128]

CFU, colony-forming unit; FBS, fasting blood glucose; HbA1c, glycated hemoglobin; OGTT, oral glucose tolerance test; HOMA-IR, homeostasis model assessment of insulin resistance; LPS, lipopolysaccharides; HDL-C, high-density lipoprotein cholesterol; IGF1R, insulin-like growth factor 1 receptor; ZO-1, zona occludens protein-1; —, Not reported; ↑, rise; ↓, decline.

**Table 3:** Clinical studies on the effect and mechanism of probiotics on diabetes.

Probiotics	Sample	Dosage	Duration	Results	Reference
*Bifidobacterium bifidum* and *Lactobacillus acidophilus*	20 patients with T2DM	1 × 10^8^ CFU/mL	2 weeks	HDL-C ↑ Fasting glycemia ↓	Moroti et al. [129]
*Lactobacillus paracasei* HII01	50 patients with T2DM	50 × 10^9^ CFU/d	12 weeks	FBS, LPS, TNF-α, IL-6 and hsCRP ↓	Toejing et al. [14]
*Lactobacillus acidophilus*, *Lactobacillus casei*, and *Bifidobacterium bifidum*	60 patients with TDM	2 × 10^9^ CFU	12 weeks	Blood glucose and insulin sensitivity ↓	Soleimain et al. [130]
*Lactobacillus acidophilus*, *Lactobacillus casei* and *Lactobacillus rhamnosus*	54 patients with T2DM	1 × 10^9^ CFU/mL	8 weeks	TGL and HOMA-IR plasma levels ↑ Serum CRP ↓	Asemi et al. [114]
*Lactobacillus acidophilus*	136 patients with T2DM	1 × 10^8^ CFU	12 weeks	Blood glucose ↓ Activity of antioxidant enzymes ↑	Mirmiranpour et al. [131]
*Lactobacillus reuteri* DSM 17938	46 patients with T2DM	1 × 10^10^ CFU/d	12 weeks	FBS, HbA1c, insulin, TC, TG, LDL-C, CRP ↓ HDL-C ↑	Mobini et al. [132]
*Lactobacillus sporogenes*	81 patients with T2DM	1 × 10^8^ CFU	8 weeks	Serum insulin levels ↓	Tajadadi et al. [133]

hsCRP, high-sensitivity c-reactive protein; TGL, total glutathione level; CRP, C-reactive protein; TC, total cholesterol; TG, triglycerides. ↑, rise; ↓, decline.

## 6. Future Development Prospect of Probiotics to Regulate Blood Glucose

There is an increasing interest in the study of probiotic regulation of blood glucose. Regulation of gut microbiology by probiotics is a potential mechanism. According to the different properties of probiotics, it is theoretically feasible to select different and appropriate combinations to regulate gut microecology in diabetic patients. With the research on the relationship between gut microbes, obesity, and T2DM, we need to focus on the future screening of potential hypoglycemic probiotics, the development of synthetic biology, the utilization of the next generation of probiotics, and the application of postbiotics and paraprobiotics.

### 6.1. Screening of Potential Hypoglycemic Probiotics

The screening probiotics need to meet the following three core characteristics: safe and harmless and have a healthy effect on the body, maintain a viable state of bacteria, and have sufficient quantities. At present, there are two main models for the isolation and identification of probiotics: (1) In vitro screening model of Caco-2 cells: simulates intestinal transport in vivo to screen probiotics with excellent performance rapidly and analyzes the bacterial strain’s adhesion to the intestine and its impact on intestinal barrier function; (2) Mouse digestive model by gavage: dietary intervention (i.e., adding probiotics to food) was carried out with the help of sterile mouse model and the function of probiotics was explained through the apparent changes in mice [134,135]. The relevant research contents mainly focused on anti-tumor, anti-cancer, diabetes prevention, intestinal inflammation treatment, and obesity prevention. Wang et al. [136] took the world’s characteristic foodborne substances as the source of lactic acid bacteria and determined the α-glucosidase inhibition ability of the strain. Screened *Lactobacillus rhamnosus* LB1lac10 had the effect of lowering blood glucose. At the same time, it was determined that the exopolysaccharide extracellular polysaccharide (EPS1-1) produced by this strain may also act as a natural α-glycosidase inhibitor to regulate blood glucose concentration, and this strain and its exopolysaccharide have particular potential in the development of hypoglycemic foods in the future.

The rapid development of high-throughput sequencing technology in recent years has made it possible to conduct an in-depth analysis of complex samples of traditional fermented food, soil, feces, and so on, filling the data missing caused by technical defects, and promoting the comprehensive analysis of microbial community structure characteristics, functional genes, metabolic pathways, and other information. This lays a foundation for the efficient screening and development of probiotics and then provides new ideas and methods for the exploration and development of beneficial microorganisms, especially those regulating blood glucose [137,138,139].

### 6.2. Development of Synthetic Biology

In recent years, with the development of synthetic biology, the next generation of microbial therapies focuses on transforming probiotics into “drug synthesis factories” that can autonomously replicate and detect abnormal conditions to synthesize and release therapeutic factors in the human body. The engineering transformation of natural probiotics and the application of the obtained engineering probiotics to disease monitoring and targeted therapy is a very effective and feasible strategy with a broad application prospect. The therapeutic advantages of engineered probiotics are apparent, such as low cost, few side effects, and simple treatment mode [140]. Studies have shown that engineered symbiotic bacteria can reprogram intestinal cells into glucose-responsive insulin-secreting cells to treat diabetes [141]. Zhang et al. [142] constructed optogenetically regulated engineered *Lactobacillus* by synthetic biology to achieve controlled secretion of GLP-1 in the organism’s intestine under in vitro blue-light stimulation, thus exerting a role in regulating blood glucose. At present, medicine is moving towards the stage of individualized treatment, and probiotics as carriers can play a pivotal role in this field [143]. In the future, each probiotic could be used as a tailored biological therapy based on a patient’s specific clinical situation [144]. With the continuous improvement and development of synthetic biology and other technologies, the exploration of probiotics will have more significant breakthroughs in the field of food and biomedicine.

### 6.3. Next-Generation Probiotics

Next-generation probiotics refer to microbial genera and species that have never been used in the food industry. Candidates have been searched for in health-related gut bacteria, including strains of the genera *Enterococcus faecalis*, *Clostridium*, *Bacteroides*, and *Ackermannia*, as well as genetically modified strains (usually *Lactococcus lactis* with novel health-beneficial properties) [145,146]. In recent years, with the gradual deepening of research, the next generation of probiotics began to appear as a new prevention and treatment tool, and it is expected to provide a potential targeted pathway and a new direction for the prevention and treatment of diseases. *Akkermansia muciniphila* has been extensively studied in the treatment of metabolic disorders, which can improve insulin resistance and intestinal permeability, increase the energy consumption of obese mice after pasteurization, and thus alleviate diabetes [147,148]. *Bifidobacterium harzianum* improved insulin sensitivity, increased energy expenditure, increased butyrate production, and regulated gut microbiota composition in diabetic mice [149]. *Pseudomonas hominis*, *Caulobacter* spp., and *Pseudomonas* spp. can prevent metabolic disorders and obesity by reducing serum leptin levels and fasting blood glucose concentration and improving glucose tolerance. They are considered potential therapeutic targets for T2DM patients [150,151]. *Prevotella copr* can produce succinic acid in the TCA circulation to improve prediabetic syndrome [152,153].

Compared with traditional probiotics, the prominent function of next-generation probiotics is therapeutic, which is worthy of further exploration by researchers. However, the relevant experiments still remain at the level of animal testing and need to be verified in human trials. In the future, the next-generation probiotics are expected to be utilized to develop specific strains of bacteria that can treat diabetes or transplant the relevant strains into the intestinal tract. We hope to achieve better therapeutic effects and provide a new, safer, healthier, and more effective way to treat diabetes.

### 6.4. Postbiotics and Paraprobiotics

Probiotics are “living microbes” that can provide health benefits to their hosts. But their inactive ingredients are called paraprobiotics, which are more effective alternatives for susceptible individuals to use [53]. Postbiotics are defined as “inactivated bacteria and bacterial components that have a beneficial effect on the host”. They include cellular components, secreted materials, metabolites, and non-viable microorganisms, which play vital roles in restoring gut microbiota [154]. Due to their potential to replace antibiotics, post-biologics have been widely used in general food, health food, and gastrointestinal therapy [155]. Studies have shown that postbiotics can improve insulin sensitivity, reduce blood sugar levels, and shorten the course of diabetes [156]. Therefore, postbiotics and paraprobiotics have recently been used as better alternatives for the treatment and prevention of metabolic diseases and their complications. These are also the directions we need to study in the future.

## 7. Current Challenges in the Regulation of Blood Glucose by Probiotics

At present, there are some limitations in the clinical application of these novel live microbial therapies, and there are some concerns about their use in patients with immune dysfunction, intestinal barrier dysfunction, and newborns [157]. Given that probiotics are living microorganisms, many biological and biopharmaceutical barriers limit their clinical application [158]. The complexity of intestinal flora, the clinical application evaluation of novel therapeutic methods, and the lack of big clinical data all require further exploration and research in the future [159]. Although some probiotics have achieved good results in vitro and in vivo, research on genetic information, genetic stability, and safety of probiotics is not in-depth. Clinical promotion and wide application are still limited, and more research is needed to understand the impact of gut microbiota on the development of diabetes.

The results of this study can prove that probiotics improve diabetes, but this may fluctuate due to different strains and individual differences [160,161]. At the same time, probiotics may also be resistant to some experimental subjects or have other unfavorable effects, which need to be comprehensively considered before conducting a comprehensive study on specific probiotic strains.

In addition, although some progress has been made in the research on the regulation of blood glucose by probiotics in recent years, the research on how probiotics play a role and its detailed mechanism is still not perfect. Furthermore, the research on the regulation of blood glucose by targeted probiotics is still not perfect. Therefore, further studies at the genetic and molecular levels are needed to develop probiotic products with solid targeting and precise regulation of blood glucose.

## 8. Conclusions

As a potential new intervention target for the treatment of diabetes, probiotics may participate in the regulation of energy metabolism through various mechanisms, namely, reducing chronic low-grade inflammation, regulating intestinal flora, increasing intestinal metabolites SCFAs, reducing oxidative stress, increasing bacterial bioactive peptides and improving insulin resistance, to achieve the purpose of regulating blood glucose. Probiotics are considered economical and safe alternatives to treat chronic diseases and improve human health. However, the prevention and alleviation of chronic diseases such as hyperglycemia through probiotics and their preparations is often a comprehensive synergistic effect of multi-factors, multi-links, multi-sites, and multi-mechanisms. The research on the mechanism of probiotics in lowering blood glucose levels and the interaction between probiotics and a variety of active substances is still insufficient. In the future, it is still necessary to strengthen the basic theory related to probiotics and the mechanism of action at the cellular and molecular levels. Although there are some deficiencies in probiotics at present, these deficiencies will be improved with the deepening of research and the development of science and technology. Therefore, the development of probiotics with blood glucose regulation function and related functional foods is of great significance for the development of the probiotic industry.

## Figures and Tables

**Table 1:** Probiotics and their main functions.

Bacterial Genus	Probiotics	Main Functions	Reference
*Bifidobacterium*	*Bifidobacterium infantis*	Prevent constipation, regulate blood glucose, inhibit intestinal pathogens, regulate intestinal balance, reduce cholesterol, promote the digestion and absorption of nutrients, delay aging, and enhance the body’s immune activity.	[16,17,18]
	*Bifidobacterium longum*		
	*Bifidobacterium bifidum*		
	*Bifidobacterium breve*		
	*Bifidobacterium animalis (Bifidobacterium lactis)*		
	*Bifidobacterium adolescentis*		
*Lactobacillus*	*Lactobacillus fermentum*	Prevent diarrhea and intestinal infections, relieve inflammatory intestinal diseases, regulate blood glucose, improve insulin resistance, increase SCFA levels, inhibit the growth of pathogenic bacteria, and reduce cholesterol levels.	[19,20,21,22,23,24]
	*Lactobacillus casei*		
	*Lactobacillus plantarum*		
	*Lactobacillus rhamnosus*		
	*Lactobacillus reuteri*		
	*Lactobacillus paracasei*		
	*Lactobacillus acidophilus*		
	*Lactobacillus crispatus*		
	*Lactobacillus bulgaricus*		
	*Lactobacillus gasseri*		
	*Lactobacillus helveticus*		
	*Lactobacillus johnsonii*		
	*Lactobacillus salivarius*		
	*Lactobacillus sakei*		
*Lactococcus*	*Lactococcus Lactis* subsp. *Lactis*	Regulate immunity and produce antimicrobial substances.	[25,26]
	*Lactococcus Lactis* subsp. *Cremoris*		
	*Lactococcus Lactis* subsp. *Diacetylactis*		
*Streptococcus*	*Streptococcus thermophiles*	Regulate immunity and improve intestinal microenvironment.	[27]
*Leuconostoc*	*Leuconostoc mesenteroides* subsp. *Mesenteroides*	Regulate immunity, inhibit harmful bacteria, and improve intestinal microenvironment.	[28,29,30]
*Bacillus*	*Bacillus coagulans*	Relieve and treat diarrhea, constipation, and indigestion.	[31]
*Propionibacterium*	*Propionibacterium freudenreichii* subsp. *Shermanii*	Regulate immunity, promote intestinal flora balance, and anti-inflammatory.	[32,33]
	*Propionibacterium acidpropionici*		
*Pediococcus*	*Pediococcus acidilactici*	Enhance immunity, promote intestinal flora balance, anti-inflammatory, and inhibit pathogenic bacteria.	[34,35,36]
	*Pediococcus pentosaceus*		
*Saccharomyces*	*Kluyveromyces marxianus*	Enhance immunity, anti-inflammatory, and inhibit the growth of pathogenic bacteria.	[37,38,39]
	*Saccharomyces cerevisiae*		
	*Saccharomyces boulardii*		
*Staphylococcus*	*Staphylococcus fleurettii*	As a starter and enrich the flavor of the product.	[40,41,42]
	*Staphylococcus hominis*		
	*Staphylococcus aureus*		
	*Staphylococcus carnosus*		
	*Staphylococcus vitulinus*		

## Data Availability

No new data were created or analyzed in this study. Data sharing is not applicable to this article.
